# Vitamin D deficiency rickets – tip of the ice burg

**DOI:** 10.1186/1687-9856-2015-S1-P64

**Published:** 2015-04-28

**Authors:** Navoda Atapattu, Subashini Jayasena, Eresha Jasinghe, Dayanath Bolonnage, Shamya De Silva

**Affiliations:** 1 Lady Ridgeway Hospital, Colombo, Sri Lanka; 2 Teaching Hospital Karapitiya, Galle, Sri Lanka; 3 Faculty of Medicine, University of Colombo, Colombo, Sri Lanka

## Introduction

Vitamin D deficiency rickets is an increasingly recognized condition across the world in both developed and developing countries. There is no vitamin D fortification program in Sri Lanka. The main source of Vitamin D is sun exposure. The burden of vitamin D deficiency rickets was not studied Sri Lanka.

## Aims

Aims of this study were to identify the number of children affected with Vitamin D deficiency rickets in a single center, affected age groups and the level of vitamin D at which the nutritional rickets appears

## Method

The study was conducted in the largest children’s hospital in Sri Lanka. The children with radiological rickets were included in the study. Patients with chronic renal failure and liver failure were excluded. The study was conducted from 2012 June to 2014 June. Data obtained from the department of chemical Pathology Lady Ridgeway Hospital Sri Lanka. Vitamin D level was measured using Chemiluminescence method.

## Results

Total number of patients presented with rickets was 42.There were 36 children with vitamin D deficiency rickets (F=21). Median age of presentation was 22 months. 22 patients were below 2 years of age (6%). All patients came with complaints of bowing of legs except a 5 month old baby who presented with hypocalcaemic convulsions. The median vitamin D level was 27.9 nmol/L (range 11.5-38.3). There were 18 (50%) children with vitamin D level less than the sample median of 27.9 nmol/L.

## Conclusion

Vitamin D deficiency rickets is seen in Sri Lankan children despite the presence of sunshine throughout the year. 61% of the children were below 2 years. Lack of sun exposure due to socio economic changes happened in the recent past would have contributed to vitamin D deficiency rickets. Further community based larger studies are needed to identify the prevalence of vitamin D deficiency and factors contributing to vitamin D deficiency rickets in Sri Lanka.

